# A study of correlation of cheiloscopy, fingerprint patterns and palatoscopy in skeletal malocclusions

**DOI:** 10.21142/2523-2754-1302-2025-237

**Published:** 2025-05-16

**Authors:** Priyanka Gaba, Deepak Kumar Gupta, Litesh Singla, Kewal Krishan

**Affiliations:** 1 Department of Orthodontics and Dentofacial Orthopedics, Panjab University, Dr Harvansh Singh Judge Institute of Dental Sciences & Hospital. Chandigarh, India. pgaba78@gmail.com Punjab University Department of Orthodontics and Dentofacial Orthopedics Panjab University Dr Harvansh Singh Judge Institute of Dental Sciences & Hospital Chandigarh India; 2 Department of Orthodontics and Dentofacial Orthopedics, Panjab University, Dr Harvansh Singh Judge Institute of Dental Sciences & Hospital. Chandigarh, India. MDS (Orthodontics and dentofacial orthopedics) drdeepak@pu.ac.in, drlitesh@yahoo.com Punjab University Department of Orthodontics and Dentofacial Orthopedics Panjab University Dr Harvansh Singh Judge Institute of Dental Sciences & Hospital Chandigarh India; 3 Department of Anthropology, Panjab University. Chandigarh, India. PhD, FRAI, FIALFS, FIACME. gargkk@yahoo.com Punjab University Department of Anthropology Panjab University Chandigarh India gargkk@yahoo.com

**Keywords:** chieloscopy, dermatoglyphics, palatoscopy, skeletal malocclusion, quieloscopia, dermatoglifos, palatoscopia, maloclusión esquelética

## Abstract

**Objective:**

For early diagnosis of malocclusion various methods such as lip prints, fingerprints and palatal rugae have been studied in the past, however, this study was unique in that it takes into consideration three factors i.e., lip prints, fingerprints and palatal rugae simultaneously to correlate with malocclusion.

**Materials and Methods:**

105 participants were equally divided as class I, class II and class III malocclusion based on ANB angle, Beta angle and Wits appraisal. The lip prints were recorded using lipstick cellophane method and were examined by Suzuki and Tsuchihashi method. For recording fingerprints, ink and stamp method was used and were analysed using Michael and Kucken method. Palatal rugae were marked on patients’ maxillary casts and examined using the Lysell and the Kapali et al classification. To investigate the relationship between lip prints, fingerprints, palatal rugae, and skeletal malocclusions, the aforementioned values were all put through the Chi-square test.

**Results:**

The lip print analysis revealed type II pattern being most predominant in all malocclusion groups. The fingerprint pattern analysis revealed Loop pattern being most predominant and arch pattern as least predominant. Whorl pattern was more frequent in Class III malocclusion compared to class I and class II malocclusion. The palatal rugae pattern revealed curved shape of palatal rugae and primary rugae as most predominant in all malocclusion groups.

**Conclusion:**

The study may have implications in allometric evaluations pertaining to anthropological, anatomical, morphological studies, however, the study may not be directly useful in early diagnosis of skeletal malocclusion.

## RESUMEN

**Objetivo:**

Para el diagnóstico temprano de la maloclusión, se han estudiado en el pasado varios métodos, como las huellas de labios, las huellas dactilares y las rugas palatinas; sin embargo, este estudio fue único porque tiene en cuenta tres factores: las huellas de labios, las huellas dactilares y las rugas palatinas simultáneamente para correlacionarlas con la maloclusión.

**Materiales y métodos:**

105 participantes se dividieron equitativamente en maloclusiones de clase I, clase II y clase III, según el ángulo ANB, el ángulo beta y la evaluación del ingenio. Las huellas de labios se registraron utilizando el método de celofán de lápiz labial y se examinaron mediante el método de Suzuki y Tsuchihashi. Para registrar las huellas dactilares, se utilizó el método de tinta y sello, y se analizaron utilizando el método de Michael y Kucken. Las rugas palatinas se marcaron en los modelos maxilares de los pacientes y se examinaron utilizando las clasificaciones de Lysell y Kapali et al. Para investigar la relación entre las huellas de labios, las huellas dactilares, las rugas palatinas y las maloclusiones esqueléticas, todos los valores antes mencionados se sometieron a la prueba de chi-cuadrado.

**Resultados:**

El análisis de la huella labial reveló que el patrón tipo II era el más predominante en todos los grupos de maloclusiones. El análisis del patrón de huellas dactilares reveló que el patrón de asas era el más predominante y el patrón de arco el menos predominante. El patrón de verticilos fue más frecuente en la maloclusión de clase III en comparación con las maloclusiones de clase I y II. El patrón de rugas palatinas reveló que la forma curva de las rugas palatinas y las rugas primarias era la más predominante en todos los grupos de maloclusiones.

**Conclusión:**

El estudio puede tener implicaciones en las evaluaciones alométricas relacionadas con los estudios antropológicos, anatómicos y morfológicos; sin embargo, el estudio puede no ser directamente útil en el diagnóstico temprano de la maloclusión esquelética.

## INTRODUCTION

The study of skin patterns and fingerprints is known as dermatoglyphics. By the 24th week of intrauterine life, they are fully formed and start to show signs during the 12th week. It is stated that these configurations hold constant for the duration of an individual's life after that^1^. The lip, alveolus, palate, and prints on the fingers and palms all grow during same embryonic period. Therefore, variations in the appearance of finger and palm prints may also come from any event generating alterations in the lip, alveolus, and palate^1^. In order to identify early malocclusion, fingerprint patterns and other characteristics of dermal ridges may provide clear benefits and be employed as a screening method that is affordable, conveniently accessible, and non-invasive.^1^

The term "dermatoglyphics," which refers to the study of the complex skin patterns and ridge formation from the palms, soles, fingers, and toes, was first used in 1926 by Cummins and Midlo. ^(^^2^These patterns are exclusive to each person and don't change until death. Even the fingerprints of monozygotic twins differ from one another. ^(^^3^Dermatoglyphics has demonstrated its usefulness in anthropology, cytogenetic research, and criminology. ^(^^3^The most effective and popular technique for personal identification, both postmortem and antemortem, is forensic medicine^4^.

In the field of dentistry, patients with periodontitis^5^, dental caries^6^^,^^7^, and certain congenital anomalies like cleft lip and palate^8^^,^^9^, have been found to have irregular fingerprints. More recently, dermatoglyphics has been linked to malocclusion^10^^,^^11^, and other developmental abnormalities of the orofacial structures. Malocclusion is one of the most common oral conditions that affect facial aesthetics. It can involve malaligned teeth, incorrect jaw positioning, or a combination of both. Any abnormality in the genome may reflect in these derivatives of the dermal ridge, which may then help as a diagnostic adjunct for malocclusions very early in life. ^(^^12^^,^^13^However, due to a lack of understanding, the idea of using fingerprints to determine malocclusion is not very common. Because the teeth, palate, and dermal patterns grow at the same time, dermatoglyphics and dental occlusion are intimately related.

In the same manner, each person's lip prints are distinct. Cheiloscopy is the study of the creases and grooves on the labial mucosa, often known as lip prints. The first person to research lip prints was Fischer, an anthropologist who worked in 1902. Le Moyne Snyder^14^was the first to propose the use of lip prints as a form of identification. They can be applied to cytogenetic research, criminal investigations, and personal identification. 

Palatoscopy, often referred to as palatal rugoscopy, is the study of a person's palate and rugae in order to ascertain their identification. Palatal rugae are asymmetric, uneven ridges of mucous membrane that stretch laterally from the anterior region of the median palatal raphe and the incisive papilla. Lund (1924) ^(^^15^noted that the palate's stratum reticulum and submucosal fatty tissue are firmly entrenched in a connective tissue core. This centre serves as a base upon which the rugae's material grows to form a fold-like projection in the roof of the mouth. According to Hausser E. Zur Bedeutung (1951) ^(^^15^, the length and spacing between the rugae increase as the anterior region of the palate grows larger during the early years of life. Throughout life, the rugae's orientation pattern doesn't change. There are anywhere from three to five rugae on either side of the palate. Once developed, they remain in the same place for the duration of an individual's life, with the exception of normal growth-related alterations in length. Trauma, chemical aggression, or illness don't seem to be able to alter palatal rugae. It can be applied in forensics, orthodontics, and gender determination. ^(^^16^

Thus, it may be possible to identify early malocclusion using lip prints, fingerprints, and palatal rugae-all of which are stable, dependable, imitable, practical, affordable, and time-efficient modes. There have been studies conducted with each of them (lip print, fingerprint and palatal rugae pattern) individually to find correlation with skeletal malocclusions, however the current study aims to correlate all three of them (lip prints, finger prints, and rugae pattern) simultaneously with all skeletal malocclusions (Class I, class II, class III), i.e, to find correlation between cheiloscopy, fingerprint pattern, and palatoscopy and skeletal class I, class II, and class III malocclusion and to possibly include cheiloscopy, fingerprint pattern assessment and palatoscopy in routine orthodontic investigation procedures as an adjunct procedure in individual identification.

## MATERIALS AND METHODS

This study was conducted in the Department of Orthodontics and Dentofacial Orthopedics of Dr. Harvansh Singh Judge Institute of Dental Sciences & Hospital, Panjab University Chandigarh. The ethical clearance for the current study was obtained from the Institutional Ethics Committee of Panjab University, Chandigarh (approval no: PUIEC220519-II-092). Study design was cross-sectional with a sample size of 105 patients undergoing orthodontic treatment.

Inclusion criteria involved, (i) Healthy individuals with well-defined rugae, (ii) Participants with unchapped lips and, (iii) Non growing patient as diagnosed with CVMI (cervical vertebral maturation index) stage of 5 or 6, or patient in the age range of 18 years and above were studied. Exclusion criteria were, (i) Participants having lesions on the lips or any congenital facial defects, (ii) Individuals with known hypersensitivity to lipsticks and, (iii) Abnormalities such as tobacco-related disorders, denture wearers, and palatal diseases also, (iv) Exclusions also included physical impairment, systemic disease, and syndromes.

**Figure 1:**
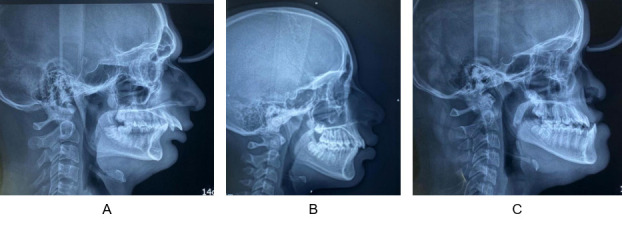
a) Skeletal Class II malocclusion b) Skeletal Class I malocclusion c) Skeletal Class III malocclusion

Lateral cephalograms of all the participants were obtained and participants were divided as class I, class II and class III malocclusion (Figure 1) based on ANB angle (cephalometric measurement for assessing saggital skeletal discrepancy) i.e., 2-4 indicates class I, >4 indicates class II and < 0 indicates class III malocclusion (also considering Beta angle and Wits appraisal).

For Cheiloscopy, the lips were first cleaned with tissue paper in order to record the lip prints. Then, a lipstick applicator was used to carefully apply lipstick in a single stroke from the centre to the lateral part of the upper lip. To guarantee that the lipstick application is consistent, the subjects were instructed to clutch both lips together. The cellophane tape's glue part was employed to take the lip impression after two minutes of waiting. The cellophane tape was carefully placed on white bond paper and this record was quickly transferred to it. Only the middle section of the lower lip was taken into consideration for analysis, and each lip print was topographically separated into six regions. Based on the numerical dominance of the line visible in the study area's patterns, the prints were analysed. The lip print was deemed indeterminate if two patterns were predominant. 

**Figure 2:**
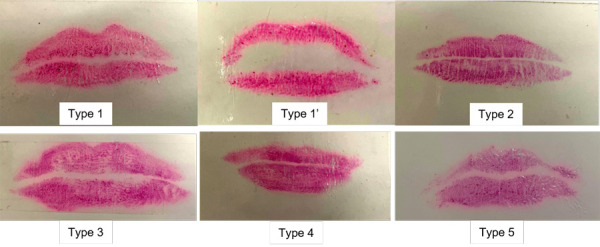
Types of Lip prints

The following procedure, which was suggested by Suzuki and Tsuchihashi^17^, was used to further categorise lip prints: - Type 1 present with distinct vertical grooves that run across the entire lip; Type 1´ is similar to type 1, but do not extend the entire lip surface; Type 2 are the branched grooves; Type 3 show intersected grooves; Type 4 present with Reticular grooves; and Type 5 include grooves that cannot be determined morphologically (Figure 2).

**Figure 3:**
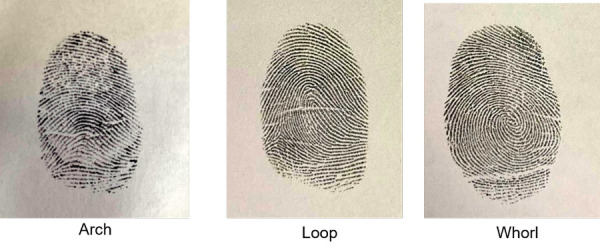
Different types of fingerprint patterns taken in the study

For Finger print pattern, using an ink pad, the left thumb imprint was obtained. It was then transferred to white bond paper and examined under a magnification lens. The most commonly used Michael and Kucken classification^18^-which divides finger print patterns into arch-like, loop, whorl and composite categories (Figure 3) was used to analyse the fingerprints.

**Figure 4:**
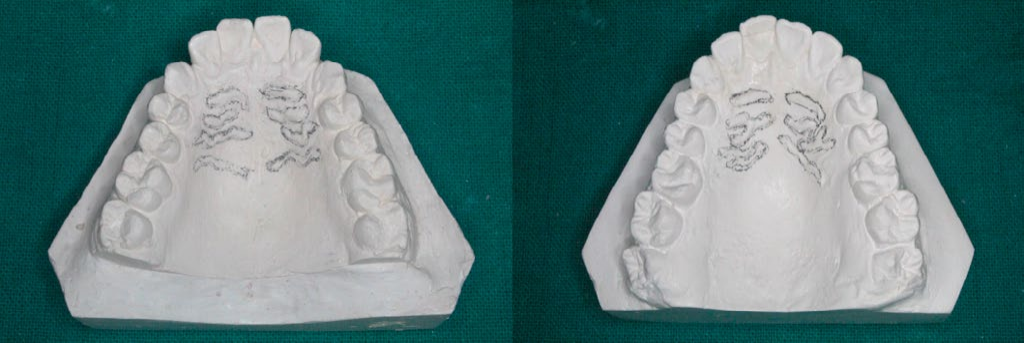
Rugae marked with lead pencil on casts

For Palatoscopy, the rugae region was the focus of the impression of the maxillary arch. After pouring the cast, the rugae area was marked with a lead pencil (Figure 4). The length of each rugae from one end to the other was measured using a divider and a scale to determine its size. 

Lysell classification: Depending on the palatal rugae's length, it is categorised into: Primary rugae > 5 mm, Secondary rugae 3-5 mm, Fragmentary rugae 2-3 mm, Smaller than 2mm in length were discarded. Shapes of rugae were analysed using Kapali et al. classification^19^. The rugae are classified into four groups according to their shapes: Wavy -If there is a slight curve at the origin or termination of curved rugae, Circular- Rugae that form a definite continuous ring are classified as circular, Straight -They run directly from their origin to termination, Curved- They are crescent shape and curved gently.

### Statistical analysis

The statistical software SPSS 26.0 (SPSS Inc., Chicago, IL) was utilised for data analysis, with a significance level of p<0.05. To evaluate the mean and standard deviation of each group, descriptive statistics were used. The Shapiro-Wilkinson test was used to determine normality of the data. The Chi square test was used in inferential statistics to determine the association between the groups. Descriptive statistics of same are mentioned in Tables 1-4.

## RESULTS

‘Lip Print Pattern’ between different malocclusion groups analysed by Chi square test reported significant difference in frequency of various patterns (Type 1/Type 1’/Type 2/Type 3/Type 4/Type 5) (P<0.05). Among the five pattern, ‘Type 2’ pattern was found to be highest in frequency in all the three malocclusion types and ‘Type 4’ pattern was reported to be the least frequent type (Table 1).

**Table 1:** Comparison of Lip Print Patterns Between Different Malocclusion Groups

Pattern	CLASS I (n=35)	CLASS II (n=35)	CLASS III (n=35)	CHI SQUARE	P VALUE
Type 1	6 (17.2%)	3 (8.6%)	1 (2.9%)	36.22	0.0001*
Type 1’	4 (11.4%)	2 (5.7%)	1 (2.9%)		
Type 2	15 (42.9%)	15 (42.9%)	23 (65.7%)		
Type 3	6 (17.2%)	7 (20%)	3 (8.6%)		
Type 4	1 (2.9%)	1 (2.9%)	3 (8.6%)		
Type 5	3 (8.6%)	7 (20%)	4 (11.4%)		

‘Finger Print Pattern’ between different malocclusion groups analysed by Chi square test did not report significant difference in frequency of various patterns (Arch/Loop/Whorl) (P>0.05). Among the three pattern, ‘loop’ pattern found to be highest in frequency in all the three malocclusion types and ‘arch’ pattern reported to be the least frequent type (Table 2). 

**Table 2:** Comparison of Finger Print Patterns Between Different Malocclusion Groups

Pattern	CLASS I (n=35)	CLASS II (n=35)	CLASS III (n=35)	CHI SQUARE	P VALUE
Arch	1 (2.9%)	2 (5.7%)	2 (5.7%)	6.67	0.08
Loop	22 (62.8%)	23 (65.7%)	17 (48.6%)		
Whorl	12 (34.3%)	10 (28.6%)	16 (45.8%)		

‘Palatal Rugae -Shape’ between different malocclusion groups analysed by Chi square test reported significant difference in frequency of various patterns on right side (Wavy/Circular/Straight/Curved) (P<0.05). Similar significance was not reported with respect to left side (P>0.05). Among the four pattern, ‘Curved’ pattern was found to be highest in frequency in all the three malocclusion types and ‘Circular’ pattern reported to be the least frequent type (Table 3). 

**Table 3:** Comparison of Palatal Rugae - Shape Between Different Malocclusion Groups

	Palatal Rugae Shape	CLASS I	CLASS II	CLASS III	CHI SQUARE	P VALUE
RIGHT	Wavy	2 (5.7%)	3 (8.6%)	0	13.92	0.03*
	Circular	0	0	0		
	Straight	3 (8.6%)	1 (2.9%)	1 (2.9%)		
	Curved	30 (85.7%)	31 (88.6%)	34 (97.1%)		
LEFT	Wavy	3 (8.6%)	2 (5.7%)	3 (8.6%)	10.02	0.12
	Circular	0	0	0		
	Straight	1 (2.9%)	4 (11.4%)	1 (2.9%)		
	Curved	31 (88.6%)	29 (82.8%)	31 (88.6%)		

‘Palatal Rugae -Size’ between different malocclusion groups analysed by Chi square test did not report significant difference in frequency of various size on both right & left side (Primary/ Secondary/Fragmentary) (P>0.05). Among the three size ‘Primary rugae’ was found to be highest in frequency in all the three malocclusion types and ‘Fragmentary rugae’ pattern reported to be the least frequent type in malocclusion groups except in class II group on left side in which ‘Secondary’ found to be the least frequent (Table 4).

**Table 4:** Comparison of Palatal Rugae - Size Between Different Malocclusion Groups

	Palatal Rugae Size	CLASS I	CLASS II	CLASS III	CHI SQUARE	P VALUE
RIGHT	PRIMARY	35 (100%)	35 (100%)	35 (100%)	0	0.99
	SECONDARY	0	0	0		
	FRAGMENTARY	0	0	0		
LEFT	PRIMARY	35 (100%)	34 (97.1%)	35 (100%)	6.06	0.19
	SECONDARY	0	1 (2.9%)	0		
	FRAGMENTARY	0	0	0		

P<0.05 is statistically significant

## DISCUSSION

Skeletal discrepancy-related malocclusions typically necessitate complicated single- or bi-jaw orthognathic procedures, which have the disadvantage of requiring a lengthy course of therapy and placing a psychological strain on the patient. Thus, early detection of developing malocclusion can help prevent the need for difficult surgeries by assisting with an interception. However, early detection of developing malocclusion may be difficult at times and hence, orthodontists have always tried to find indirect cues from other traits of patients which can help in this objective. For this reason, dermatoglyphics, lip prints, and palatal rugae patterns can be employed as a noninvasive, predictive method to spot the onset of malocclusion. Because finger and palm prints, the lip, alveolus, and palate develop concurrently, other organs, teeth, and jaws also go through the same developmental stage, this was the basis of co-relating malocclusion with the study of lip prints, dermatoglyphics, and palatal rugae in current study.

In the present study, sample size of the study was derived at the power of 80% and confidence interval of 95%. Calculated minimum sample size was 96. However, for each group the sample size taken was 35 to account for attrition. For recording the lip prints, lipstick- cellophane method was used. In addition to acting as the patient's permanent lip record, this technique could be safely saved for further examination. Similar to the study by Vignesh et al. (2017) ^(^^20^, each lip print was topographically separated into six parts for analysis, with just the middle portion of the lower lip taken into consideration due to simplicity of visualisation. The approach for classifying lip prints that was suggested by Suzuki and Tsuchihashi was adhered to, as it has been widely recognised and employed in the majority of previous investigations.

In order to record fingerprints, the left thumb's impression was produced using the ink and stamp procedure. Thumb impressions from the left hand are frequently used for identification verification for a variety of reasons, and they are seen to be a dependable and consistent approach for the same. The finger print pattern was analysed using the most commonly used classification system, Michael and Kucken, which divides finger print patterns into loop, whorl, arch-like, and composite patterns. 

To record palatal rugae, patients’ maxillary casts were used. On these casts, rugae were drawn with a sharp lead pencil. The magnifying glass was then used to examine the palatal rugae pattern on these casts. This method was employed in most of the studies conducted previously to record palatal rugae. Since analysing the pattern was more significant for us than determining the quantity, direction, and unification of rugae, we used the most widely recognised classification systems: Lysell classification to examine the length and Kapali et al. classification, to examine the shape of the rugae. To investigate the relationship between lip prints, finger prints, palatal rugae and class I, class II, and class III skeletal malocclusion, the aforementioned values were all put under the Chi-square test.

In the current study, 105 individuals in total had their palatal rugae, lip prints, and fingerprint patterns examined in relation to skeletal malocclusions. Based on cephalometric measures, ANB angle i.e., 2-4 indicates class I, >4 indicates class II and < 0 indicates class III malocclusion (also considering beta angle and wits appraisal), the 105 samples were classified as class I, class II, and class III skeletal malocclusion since ANB angle, beta angle and wits appraisal are standard criteria to classify skeletal malocclusions.

### Lip print patterns

In class I malocclusion, the branched lip pattern was the most frequent (42.9%) followed by the vertical pattern (17.2%) and intersected pattern (17.2%), undetermined pattern (8.6%), and reticular pattern (2.9%) and in class II malocclusion, the branched lip pattern was the most frequent (42.9%) followed by the intersected pattern (20%) and undetermined pattern (20%), vertical pattern (8.6%) and reticular pattern (2.9%). In class III malocclusion, the branched pattern was the most frequent (65.7%) followed by undetermined pattern (11.4%), intersected pattern (8.6%) and reticular pattern (8.6%), and vertical pattern (2.9%).

In study by Kulkarni et al (2012) ^(^^21^It was found that the skeletal class I group of people exhibited a predominance of 1,3; 1´,3; and 2,3 lip print patterns. Patients in the skeletal class III group exhibited a predominance of 1,4, and 3,4 lip print combinations. A higher prevalence of the 1, 2 type of lip print combination was found in skeletal class II individuals. The results of this study cannot be compared with current study since lips were divided into four quadrants in study by Kulkarni et al whereas middle portion of lower lip was considered in the current study. In study conducted by Aditi et al (2019) ^(^^22^, results were inconsistent with the current study as in current study branched pattern was predominant in all three groups whereas in study by Aditi et al in skeletal Class I, PVG (partial vertical groove) lip pattern was most prevalent, whereas in skeletal Class II Division 1, intersecting groove, in skeletal Class II Division 2, PVG, and in skeletal Class III, complete vertical groove were prevalent.

### Fingerprint patterns

In the current study, the loop pattern predominated in all three groups; however, the frequency of whorl pattern was higher in class III malocclusion compared to class I and class II malocclusion. The loop pattern repeated most frequently in the study by George et al (2017) ^(^^23^except class II malocclusion, in which there was an increased distribution of the whorl pattern. This study's findings, which identified loop patterns as the most prevalent patterns, are partly comparable to this one. 

Similar outcomes were found in a study conducted by Cheeli et al (2017) ^(^^24^in which loop pattern was predominant in all the subgroups (Class I, II, III malocclusion). 

### Palatal rugae patterns

Regarding palatal rugae shape between different malocclusion group significant difference in frequency of various patterns was noted in right side, however, similar significance was not reported with respect to left side. These findings are comparable to past studies as number, shape, width, height, and location of palatal rugae vary from the left side of the palate to the right side as well as from one person to another, and are unique to every individual. Among the four pattern, curved pattern found to be highest in frequency in all the three malocclusion types and circular pattern reported to be the least frequent type in all malocclusion groups. Regarding palatal rugae size between different malocclusion groups, no significant difference in frequency of various size in both right & left side was noted. Among the three size primary rugae found to be highest in frequency in all the three malocclusion types and fragmentary rugae reported to be the least frequent type in malocclusion groups except in class II malocclusion, on left side where secondary rugae were found to be the least frequent.

According to Oral et al.'s (2017) ^(^^25^study, the most prevalent rugae pattern types across all classes (class I, class II, and class III) were wavy and curved forms. This might be attributed to racial differences among the study's participants. According to Rizwan et al. (2020) ^(^^26^, Circular and curved rugae shapes were the most prevalent in all skeletal malocclusions. Nonetheless, the current study's finding that the curved rugae design had the greatest prevalence, are partly consistent with the above studies.

Though not alone, dermal ridge patterns and craniofacial traits may be primarily influenced by genetics. Therefore, any anomaly in the phases of organ or mandibular creation may manifest itself in dermatoglyphic patterns. The aetiology of sagittal skeletal disparities is multifaceted, encompassing both environmental and hereditary variables. 

Sonic hedgehog (SHH) and Wise gene plays a critical role in palatal rugae development^27^, while the dermatoglyphic pattern is determined by the EVI1 protein, EDAR signalling in the surface ectoderm is anticipated to directly affect dermatoglyphic patterns^28^, and FGFs, bone morphogenetic proteins (BMPs) and Sonic Hedgehog (SHH) plays a critical role in lip pattern formation^29^. Also considering their inheritance pattern, compared to the non-identical twins, the identical twins displayed a higher percentage of similarities. In case of lip prints, the twins' inheritance pattern was significant but their fingerprints did not exhibit the same relevance. Actinin alpha 3 (ACTN3), ADAM metallopeptidase with thrombospondin type 1 motif 9 (ADAMTS9), fibroblast growth factor receptor 2 (FGFR2), msh homeobox 1 (MSX1), and myosin IH (MYO1H)) are the genes linked with skeletal class II malocclusion are whereas collagen type II alpha 1 chain (COL2A1), fibroblast growth factor receptor 2 (FGFR2), lysine acetyltransferase 6B (KAT6B), myosin IH (MYO1H), plexin A2 (PLXNA2), and SSX family member 2 interacting protein (SSX2IP) ^(^^30^are the genes linked with skeletal class III malocclusion. It can be concluded that different genes are responsible for various traits studied in our study i.e, lip prints, finger prints and palatal rugae and malocclusion, hence no definite correlation were found between these traits and malocclusion.

As in orthodontics there are many theories of growth such as genetic, sutural, cartilage, functional matrix theory, cybernetics and so on, thereby suggesting that not a single theory can explain the development of hard and soft tissues. Hence, based on our existing knowledge and role of various theories in explaining orofacial growth, it can be safely said that dermatoglyphics being the soft tissue prints and malocclusion being dependent on hard tissue factors, correlation of both factors in other studies can be a chance phenomenon not explainable by the same way as growth and development of orofacial complex which is complex and difficult to explain based on single line of theory or hypothesis.

## CONCLUSION

The present study evaluated the correlation between the morphological features of the body such as lip prints, fingerprints, and palatal rugae patterns with skeletal malocclusion and found a strong association between the whorl fingerprint pattern and Class III skeletal malocclusion. The study may have implications in allometric evaluations pertaining to anthropological, anatomical, morphological studies. In the current study, a weak correlation could be established between malocclusion and lip prints, fingerprints, and palatal rugae patterns, which can be attributed to multifactorial aetiology of sagittal skeletal discrepancies, including genetic and environmental factors. The current study may not be directly useful in early diagnosis of skeletal malocclusion.

## References

[1] Achalli S, Patla M, Nayak U, Soans CR (2016). Dermatoglyphics and orthodontics. Int J Orthod Rehabil.

[2] Reddy KS (2005). The essentials of forensic medicine and toxicology.

[3] Gopan G (2020). Cheiloscopy & dermatoglyphics- two sides of a coin: an orthodontic review. Int J Sci Res.

[4] Pratibha R, Abhilash PR, Sherlin HJ (2011). Conventional derma¬toglyphics - revived concept a review. Int J Pharma Bio Sci.

[5] Atasu M, Kuru B, Firatli E, Meric H (2005). Dermatoglyphic findings in periodontal diseases. Int J Anthropol.

[6] Sharma A, Somani R (2009). Dermatoglyphic interpretation of dental caries and its correlation to salivary bacteria interactions an in vivo study. J Indian Soc Pedod Prev Dent.

[7] Sengupta AB, Bazmi BA, Sarkar S, Kar S, Ghosh C, Mubtasum H (2013). A cross sectional study of dermatoglyphics and dental caries in Bengalee children. J Indian Soc Pedod Prev Dent.

[8] Balgir RS (1986). Dermatoglyphic features in congenital cleft lip and cleft palate anomalies. J Indian Med Assoc.

[9] Mathew L, Hegde AM, Rai K (2005). Dermatoglyphic peculiarities in children with oral clefts. J Indian Soc Pedod Prev Dent.

[10] Reddy S, Prabhakar AR, Reddy VVS (1997). A dermatoglyphic predictive and comparative study of class I, class II, div.1, div. 2 and class III malocclusions. J Indian Soc Pedod Prev Dent.

[11] Trehan M, Kapoor DN, Tandon P, Sharma VP (2001). Dermatoglyphic study of normal occlusion and malocclusion. J Ind Orthod Soc.

[12] Bhasin MT, Bhasin P, Singh A, Bhatia N, Shewale AH, Gambhir N (2016). Dermatoglyphics and malocclusion - A forensic link. Br Biotechnol J.

[13] Kapoor P, Ragini, Kaur H (2015). Rugoscopy a diagnostic appurtenance for malocclusion or just a forensic aid? - A pilot study. J Forensic Res.

[14] Dineshshankar J, Ganapathi N, Yoithapprabhunath TR, Maheswaran T, Kumar MS, Aravindhan R (2013). Lip prints Role in forensic odontology. J Pharm Bioallied Sci.

[15] Mahajan R, Dar MA, Risam SS (2014). Palatoscopy/rugoscopy a potential tool in human identification. J Evol Med Dent Sci.

[16] Monga DK, Bhateja S, Arora G (2019). Palatoscopy A way to discover victim's identity in mass disaster. J Oral Med, Oral Surg, Oral Pathol, Oral Radiol.

[17] Tsuchihashi Y (1974). Studies on personal identification by means of lip prints. Forensic Sci.

[18] Mutalik VS, Menon A, Jayalakshmi N, Kamath A, Raghu AR (2013). Utility of cheiloscopy, rugoscopy, and dactyloscopy for human identification in a defined cohort. J Forensic Dent Sci.

[19] Kapali S, Townsend G, Richards L, Parish T (1997). Palatal rugae patterns in australian aborigines and caucasians. Aust Dent J.

[20] Ravindra V, Rekha V, Annamalai S, Sharmin D, Norouzi-Baghkomeh P (2018). A comparative evaluation between dermatoglyphic patterns and different terminal planes in primary dentition. J Clin Exp Dent.

[21] Kulkarni N, Vasudevan S, Shah R, Rao P, Balappanavar AY (2012). Cheiloscopy A new role as a marker of sagittal jaw relation. J Forensic Dent Sci.

[22] Aditi S, Tikku T, Khanna R, Maurya RP, Verma S, Srivastava K (2019). Cheiloscopy Association of lip prints in different skeletal malocclusions. Int J Orthod Rehabil.

[23] George SM, Philip B, Madathody D, Mathew M, Paul J, Dlima JP (2017). An Assessment of Correlation between Dermatoglyphic Patterns and Sagittal Skeletal Discrepancies. J Clin Diagn Res.

[24] Cheeli S, Ghanashyam Prasad M, Naga Radhakrishna A, Santosh Kumar KVK, Dangeti D, Pavanireddy S (2017). Comparative reliability of rugoscopy and dactyloscopy for the predilection of malocclusion and dental caries in children A cohort study. Pesqui Bras Odontopediatria Clin Integr.

[25] Oral E, Buyuk SK, Simsek H (2017). Evaluation of palatal rugae pattern in different sagittal skeletal relationship adolescent subjects. Medicine (Baltimore).

[26] Rizwan N, Sheikh F, Memon S, Agha D (2020). Association of Rugae Pattern with Skeletal Malocclusion in Orthodontic Patients Visiting Tertiary Care Hospital. PJMD.

[27] Hammond NL, Dixon MJ (2022). Revisiting the embryogenesis of lip and palate development. Oral Dis.

[28] Li J, Glover JD, Zhang H, Peng M, Tan J, Mallick CB, Hou D (2022). Limb development genes underlie variation in human fingerprint patterns. Cell.

[29] Mathew L, Hegde AM, Rai K (2005). Dermatoglyphic peculiarities in children with oral clefts. J Indian Soc Pedod Prev Dent.

[30] Gershater E, Li C, Ha P, Chung CH, Tanna N, Zou M, Zheng Z (2021). Genes and Pathways Associated with Skeletal Sagittal Malocclusions A Systematic Review. Int J Mol Sci.

